# Mechanical Ventilation during Extracorporeal Membrane Oxygenation in Patients with Acute Severe Respiratory Failure

**DOI:** 10.1155/2017/1783857

**Published:** 2017-01-03

**Authors:** Zhongheng Zhang, Wan-Jie Gu, Kun Chen, Hongying Ni

**Affiliations:** ^1^Department of Emergency Medicine, Sir Run-Run Shaw Hospital, Zhejiang University School of Medicine, Hangzhou 310016, China; ^2^Department of Anesthesiology, Nanjing Drum Tower Hospital, Medical College of Nanjing University, Nanjing 210008, China; ^3^Department of Critical Care Medicine, Jinhua Municipal Central Hospital, Jinhua Hospital of Zhejiang University, Zhejiang, China

## Abstract

Conventionally, a substantial number of patients with acute respiratory failure require mechanical ventilation (MV) to avert catastrophe of hypoxemia and hypercapnia. However, mechanical ventilation per se can cause lung injury, accelerating the disease progression. Extracorporeal membrane oxygenation (ECMO) provides an alternative to rescue patients with severe respiratory failure that conventional mechanical ventilation fails to maintain adequate gas exchange. The physiology behind ECMO and its interaction with MV were reviewed. Next, we discussed the timing of ECMO initiation based on the risks and benefits of ECMO. During the running of ECMO, the protective ventilation strategy can be employed without worrying about catastrophic hypoxemia and carbon dioxide retention. There is a large body of evidence showing that protective ventilation with low tidal volume, high positive end-expiratory pressure, and prone positioning can provide benefits on mortality outcome. More recently, there is an increasing popularity on the use of awake and spontaneous breathing for patients undergoing ECMO, which is thought to be beneficial in terms of rehabilitation.

## 1. Introduction

Extracorporeal membrane oxygenation (ECMO) is an important technique for the treatment of severe respiratory failure, providing opportunity for lung recovery or transplantation [1, 2]. Hill and colleagues first described ECMO support for cases of severe respiratory failure four decades ago [3]. Since then, a large number of observational studies and randomized trials have been performed [4, 5]. In common practice, ECMO is indicated when conventional mechanical ventilation fails to improve arterial oxygenation and/or eliminate carbon dioxide [6]. Another indication is the circulatory and/or cardiac failure. However, ECMO has not been well established (e.g., in the framework of evidence based medicine) for its effectiveness in the treatment severe respiratory failure, especially in some particular situations such as immune-compromised patients [7]. While there is uncertainty on the effectiveness of ECMO versus mechanical ventilation on mortality outcome, ECMO is still widely used for patients with refractory respiratory failure.

**Figure 1:**
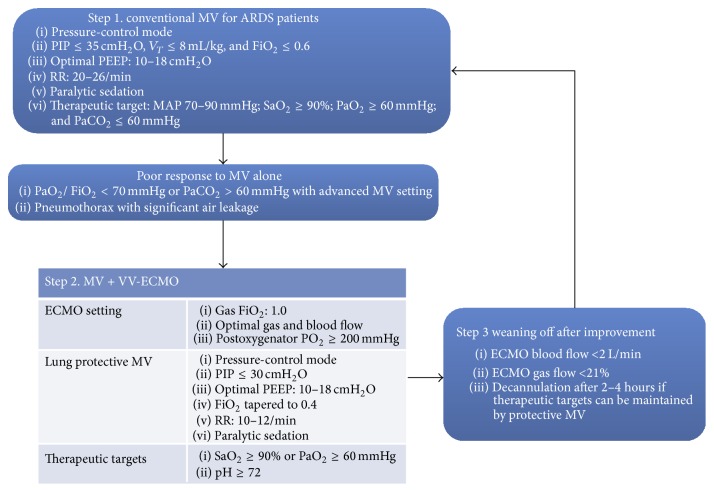
Management of severe acute respiratory distress syndrome in adults. Note that extracorporeal membrane oxygenation is provided after failure of conventional ventilation. Step  1 is the use of conventional MV for ARDS patients. Protective ventilation is typically employed. If the patient responds poorly to the initial MV setting, the strategy is to initiate VV-ECMO with the therapeutic target to maintain SaO_2_ and serum pH. Weaning off the ECMO is considered when the blood and gas flow are decreased to 2 L/min and 21%, respectively. The figure was adapted from [9] under the Creative Commons Attribution License 4.0, which permits unrestricted use, distribution, and reproduction in any medium, provided the original work is properly cited. MV: mechanical ventilation; VV-ECMO: venovenous extracorporeal membrane oxygenation; MAP: mean arterial pressure; PEEP: positive end-expiratory pressure; RR: respiratory rate.

Because ECMO is expansive, is technically challenging, and bears catastrophic complications, it is not considered as a first line therapy for patients with respiratory failure [8]. A typical therapeutic protocol of severe acute respiratory distress syndrome (ARDS) is shown in Figure 1 [9]. The first line therapy (step  1) for severe ARDS is mechanical ventilation with a variety of modes [10–13]. Protective ventilation is typically employed. If the patient responds poorly to the initial MV setting, the strategy is to initiate VV-ECMO with the therapeutic target to maintain SaO_2_ and serum pH. Weaning off the ECMO is considered when the blood and gas flow are decreased to 2 L/min and 21%, respectively [9]. During ECMO running, mechanical ventilation is still in use. As a result, respiratory support of such patients comprises the native lung and artificial lung. The mechanical ventilation setting in patients undergoing ECMO is an area of active research. There is controversy on the optimal degree of mechanical ventilation support. While ultra-protective ventilation provides enough lung rest, lung recruitment may accelerate lung recovery [14]. In the present review we summarize the current evidence on mechanical ventilation during ECMO.

## 2. Physiology behind ECMO

Because this review primarily focuses on mechanical ventilation during ECMO, we first need to understand some physiological changes during ECMO. Venovenous extracorporeal membrane oxygenation (VV-ECMO) is commonly used for the management of patients with respiratory failure and stable hemodynamics. The venous blood with low oxygen saturation (SvO_2_) is typically drained from superior vena cava, inferior vena cava, and/or large vein such as femoral or subclavian vein. It passes through the oxygenator [15] and then returns to the patient in or near the right atrium [16]. The returned blood with high oxygen content is mixed with systemic venous blood and enters into right heart. The mixed venous blood is further oxygenated in the native lung. However, due to low mechanical ventilation setting, such oxygenation is always negligible. Mechanical ventilation in this regard is more to keep the lung open than to provide oxygen [16]. However, native lung function is not always negligible; this may be the case for native lung CO_2_ removal. Respiratory drive cannot be fully controlled by extracorporeal CO_2_ removal, especially in acute hypoxemic patients.

Because ECMO is able to provide oxygen and remove carbon dioxide, the respiratory drive and effort can be controlled. A few animal studies showed that carbon dioxide removal by ECMO was able to induce apnea [17, 18]. In human study, when gas flow (e.g., control of carbon dioxide) dropped from 100% to 0%, pressure generated in the first 100 ms of inspiration against an occluded airway increased from 0.9 ± 0.5 to 2.8 ± 2.7 cmH_2_O (*p* < 0.001); the maximal inspiratory muscles pressure increased from 4.5 ± 3.1 to 8.5 ± 6.3 cmH_2_O. The authors concluded that carbon dioxide removal had significant impact on spontaneous breathing effort [19].

An important feature of VV-ECMO is its mild hemodynamic effect on circulation. This is of particular importance for hemodynamically unstable patients with acute respiratory failure (ARF). In animal models, Shen and colleagues found that although there were mild changes in ultrastructure and function of cardiomyocyte and mitochondria, the global hemodynamics were stable [20]. Also, there is evidence that the installation of VV-ECMO decreases heart rate, but mean arterial pressure is not significantly affected [21]. Given the favorable hemodynamic features of VV-ECMO, it can be used for patients with hemodynamically unstable patients. However, if a patient shows ARF in combination with refractory shock, venoarterial ECMO (VA-ECMO) should be recommended for use.

## 3. Timing of ECMO Initiation: Indications from Ventilation Parameters

Because mechanical ventilation typically precedes ECMO and mechanical ventilation parameters provide important information for the initiation of ECMO, in this section, we discuss when to start ECMO for severe respiratory failure.

The principle to start ECMO is when conventional mechanical ventilation cannot provide enough oxygenation and/or carbon dioxide elimination or ventilator setting is too high that can cause significant lung injury. Another condition is that the duration of mechanical ventilation is not too long that the underlying pathology is reversible. The timing of ECMO is usually based on the severity of ARDS, as represented by severe hypoxemia despite high PEEP (PaO_2_/FiO_2_ < 80 mmHg) and uncompensated hypercapnia (pH < 7.2) [22]. There is evidence that early initiation of ECMO (1.9 ± 1.4 days after onset of severe ARDS defined by Berlin definition) improves survival in trauma patients [23]. However, this study is limited by small sample size and the use of historical control. A large randomized controlled trial conducted by Peek and colleagues was probably the cornerstone in exploring the indications of ECMO for ARDS patients [24]. In the study, ARDS patients with Murray score > 3.0 or pH < 7.20 were randomized to receive either ECMO or conventional mechanical ventilation. The 6-month survival was 63% in the ECMO group versus 47% in the control group (*p* = 0.03). With the success of this trial, the criteria were adopted by Italian ECMO network. Use of the criteria in ARDS patients caused by influenza A (H1N1) virus showed a survival discharge rate of 68% [25]. In a well-matched cohort, early VV-ECMO was associated with lower mortality in patients with severe hypoxemic respiratory failure [26]. A threshold of plateau pressure is commonly used to avoid lung injury during mechanical ventilation. However, plateau pressure is generated by elastances of the lung and chest wall. It is the transpulmonary pressure that can cause lung injury. Grasso and colleagues reported ECMO initiation criteria using transpulmonary pressure estimated with esophageal pressure. In 14 patients with influenza A- (H1N1-) associated ARDS referred for ECMO, half of them avoided ECMO when upper limit of transpulmonary pressure equal to 25 cmH_2_O was employed [27].

There are also situations in which the use of ECMO may not be beneficial. In terms of mechanical ventilation, it was suggested that patients on mechanical ventilation for over 7 days were contraindicated for ECMO [24]. While it is well known that prolonged mechanical ventilation is a harbinger of adverse outcome, the days are not well established by empirical evidence. For example, Cheng and colleagues developed a VV-ECMO mortality score to triage patients before ECMO running, in which Pre-ECMO MV day > 4 was the most important predictor of death with a coefficient of 2 (i.e., other predictors had coefficient of 1) [28]. Other observational studies also identified similar relationship between Pre-ECMO MV days and mortality outcome [9, 29–31]. Most importantly, MV prior to initiation of ECMO is an important component in the calculation of Respiratory ECMO Survival Prediction (RESP) score. This score has been validated to assist prediction of survival for adult patients undergoing ECMO for respiratory failure [4, 32]. However, it is still difficult to determine a specific time point after which the initiation of ECMO can be considered futile. Probably, this is dependent on the sophistication of individual centers, and here individualized selection of patients should be performed.

## 4. Protective Ventilation in ECMO

It is well understood that conventional ventilation mode can cause ventilator induced lung injury (VILI). The underlying mechanisms of VILI include alveolar overdistension (volutrauma), alveolar instability leading to alveolar collapse and reopening with each breath (atelectrauma), and the secondary inflammation caused by these mechanical injuries which is known as biotrauma [33]. Volutrauma is caused by ventilation at high tidal volumes. The effect of ventilation volumes on injury is independent of the peak airway pressure. Rat models have shown that, at the same peak airway pressure (45 cmH_2_O), those ventilated with low tidal volumes developed less severe permeability and pulmonary edema [34]. In clinical practice, ventilation at high airway pressure is observed to cause lung injury manifested as pneumothorax or subcutaneous emphysema. However, since the high airway pressures per se do not cause VILI unless they are associated with high lung volumes, the term barotrauma is a misnomer [35]. To ameliorate the VILI, the concept of protective MV is introduced into clinical practice. The following paragraphs examine the use of protective ventilation in patients undergoing ECMO.

Protective ventilation with low tidal volume has long been known as a major component of ventilation strategy for both injured and healthy lung [10, 36, 37]. A landmark study on low tidal volume ventilation was conducted nearly two decades ago [38]. The study showed that patients who received protective ventilation versus conventional group had significantly lower 28-day mortality rate (38% versus 71%; *p* < 0.001). A recent network meta-analysis showed that ventilation with low tidal volume plus prone position was associated with reduced risk of death (hazards ratio: 0.62; 95% CI: 0.42–0.98) [39]. However, some studies failed to identify a beneficial effect on mortality [40, 41] or the effect size is much less than that in Amato's study [42]. While the benefit of low tidal volume ventilation is to reduce lung injury, it may cause carbon dioxide retention and hypoxemia due to reduced ventilation. In other words, the balance between lung rest and working is difficult to determine. Patient population with severe ARDS is actually an extremely heterogeneous group that one size does not fit all, and the relative importance of lung rest versus metabolic demand can be different across the population. During VV-ECMO, mechanical ventilation is still required due to reasons that (1) ECMO blood flow rate is usually not enough and in hyperdynamic status a substantial proportion of blood still passed via native lung, not having gone through the artificial lung first; (2) lung should be mildly ventilated and kept open. Complete collapse of the lung may delay its recovery. There is evidence that a sufficient PEEP level is beneficial [43].

The major obstacle for performing low tidal volume ventilation is carbon dioxide retention, worsened oxygenation, and intrapulmonary shunt [44]. When tidal volume reduces below 6 mL/kg, arterial PaCO_2_ level increased remarkably and the pH value dropped below 7.2. Such a procedure for lung rest is performed at the cost of metabolic disturbances and tissue hypoxia. Fortunately, ECMO can provide an opportunity for the lung to rest while maintaining tissue oxygen supply and carbon dioxide elimination. With extracorporeal carbon dioxide removal, Ranieri and colleagues showed that tidal volume < 6 mL/kg enhanced lung protection with respect to acid-base homeostasis, cytokine secretion, and pulmonary morphology [45]. Thus, it is wise to rest the lung in severe ARDS patients who are also supported with ECMO. In an international survey on ventilator setting during ECMO, 77% of ECMO centers reported “lung rest” as the primary goal of mechanical ventilation; a tidal volume of 6 mL/kg or less was targeted in 76% centers [46]. Although there is a lack of randomized controlled trial in this topic, there is a large body of observational evidence supporting the notion that protective ventilation is associated with better outcome [47]. In Schmidt et al.'s study, protective ventilation was routinely used in high-volume ECMO centers. Higher positive end-expiratory pressure levels during the first 3 days of ECMO support were associated with lower mortality (odds ratio, 0.75; 95% CI, 0.64–0.88; *p* = 0.0006) [43]. With multivariable regression model, it was found that each one cmH_2_O increase in plateau pressure was associated with a 14.4% decrease in the odds of achieving hospital survival (95% CI = 1.75% to 25.4%, *p* = 0.027). Conversely, each one cmH_2_O increase in PEEP was associated with a 36.2% decrease in the odds of 30-day survival (95% CI = 10.8% to 54.4%, *p* = 0.009) [48]. Pandemic influenza A is a tragedy for human being, but it provides a good opportunity for exploring mechanical ventilator setting in ECMO patients [49]. Survivors had significantly lower plateau pressure during ECMO than nonsurvivors (25 ± 3 versus 29 ± 5 cmH_2_O; *p* < 0.01). The result remained unchanged even after multivariable adjustment (OR: 1.33; 95% CI: 1.14–1.59; *p* < 0.01). More recently, some authors also explored the use of ultra-protective ventilation (i.e., tidal volume reduced to 4 mL/kg predicted body weight while PEEP was increased to target a plateau pressure between 23 and 25 cmH_2_O) with the help of low-flow extracorporeal carbon dioxide removal (ECCO_2_R) in moderate ARDS [50].

Another component of protective ventilation is low respiratory rate [51]. The rationale of this procedure is to rest the lung by reducing its motion. The lungs were ventilated 3 to 5 times per minute, with peak airway pressure limited to 35 to 45 cmH_2_O. A continuous oxygen flow was provided. Carbon dioxide elimination was performed by extracorporeal method [51].

Closed-loop ventilation represents another novel protective ventilation mode [52]. It automatically adjusts some settings according to physiological target made by physicians, making it possible to select an individualized ventilator setting [53]. IntelliVent-ASV™ is an extension and development of adaptive support ventilation (ASV) that automatically adjusts ventilation settings such as minute volume, tidal volume (VT), and respiratory rate (RR), to reach a target end-tidal CO_2_ (PETCO_2_) in passively breathing patients and a target RR in actively breathing patients. Furthermore, inspiratory fraction of oxygen (FiO_2_) and positive end-expiratory pressure (PEEP) are adjusted automatically to reach a target pulse oximetry (SpO_2_). Although the closed-loop ventilation mode has been shown to be safe and effective in patients with ARDS, its use in patients undergoing ECMO has not been fully investigated [54, 55]. In a case series involving six patients, Karagiannidis and colleagues reported that closed-loop ventilation mode responded rapidly to decreased ECMO sweep gas flow. It concluded that the combination of neurally adjusted ventilatory assist (NAVA) and ECMO might permit a closed-loop ventilation with automated protective ventilation [56].

## 5. Recruitment Maneuvers

Recruitment maneuver is the indispensable component of protective ventilation, and there are a variety of methods to perform recruitment maneuver. In this section, we aimed to describe some commonly used recruitment maneuvers. Grasso and colleagues proposed the titration of PEEP according to stress index. Stress index (*b*) can be estimated based on airway pressure and inspiratory time by the following equation:

(1) $$\begin{array}{c} \text{Airway pressure}=a\cdot {\text{Inspiratory time}}^{b}+c, \end{array}$$

where the coefficient *b* is the stress index describing the shape of the airway opening pressure (Pao) corresponding to the period of constant-flow inflation. For *b* < 1, the Pao curve presents a downward concavity, suggesting a continuous decrease in elastance during constant-flow inflation. For *b* > 1, the curve presents an upward concavity suggesting a continuous increase in elastance. For *b* = 1, the curve is straight, suggesting the absence of tidal variations in elastance. PEEP level was titrated to target a stress index between 0.9 and 1.1 [57]. Specifically, PEEP was decreased if the stress index was higher than 1.1 and was increased if the stress index was lower than 0.9. PEEP is not changed if the stress index was between 0.9 and 1.1 [58].

Talmor and colleagues proposed to set PEEP levels in reference to the esophageal pressure. Patients underwent heavy sedation and paralysis. Recruitment maneuver was performed by increasing airway pressure to 40 cmH_2_O for 30 seconds. Thereafter, PEEP was set to achieve a transpulmonary pressure of 0 to 10 cmH_2_O at end expiration, according to a sliding scale based on the PaO_2_ and the FiO_2_ (Table 1) [59]. Ventilator setting was adjusted in one column at a time to keep the partial pressure of arterial oxygen (PaO_2_) between 55 and 120 mmHg. Alternatively, the oxygen saturation, as measured by pulse oximeter, was kept between 88 and 98% by using the ventilator settings in one column at a time. The PEEP was set at such a level that transpulmonary pressure during end-expiratory occlusion (PLexp) stays between 0 and 10 cmH_2_O and keeps transpulmonary pressure during end-inspiratory occlusion at less than 25 cmH_2_O. Tidal volume was set at 6 mL/kg of predicted body weight. The predicted body weight was estimated using the following equation:

(2) Predicted  body  weight=50if  male,45.5  if  female+0.91×centimeters of height –152.4.

In the EXPRESS trial, “open-lung approach” was employed to treat patients with severe ARDS [60]. The ventilator procedures included pressure-control mode, targeting tidal volume of 6 mL/kg of predicted body weight, and plateau airway pressures less than 40 cmH_2_O. The recruitment maneuver included a 40-second breath-hold at an airway pressure of 40 cmH_2_O and an FIO_2_ of 1.0. Oxygenation was maintained in a target range as described previously using a slide scale of PEEP/FiO_2_ combinations (Table 2) [42].

## 6. Prone Positioning of Patients during ECMO

Prone position is an alternative or rescue therapy for patients with severe ARDS. Prone positioning may help to reduce collapse of dorsal lung segments with subsequent avoidance of alveolar overdistension of ventral lung segments. The aim is to homogenize transpulmonary pressure and reduce intrapulmonary shunt. In patients with severe ARDS, prone positioning has been proven to be beneficial in some clinical outcomes such as mortality (relative risk [RR]: 0.9; 95% CI: 0.82–0.98) [61], ratio of partial pressure of arterial oxygen to the fraction of inspired oxygen (63.0 ± 66.8 versus 44.6 ± 68.2, *p* = 0.02) [62], and ventilator-associated pneumonia (1.66 versus 2.14 episodes per 100 patients-days of intubation; *p* = 0.045) [63]. The well-known PROSEVA study is the largest multicenter study investigating the effect of prone positioning on mortality outcome. The study confirmed that early application of prolonged prone positioning sessions significantly decreased 28-day (16.0% versus 32.8%; *p* < 0.001) and 90-day mortality (23.6% versus 41.0%; *p* < 0.001) in patients with severe ARDS [64].

Prone positioning can be successfully performed during ECMO, and it is associated with improved respiratory parameters. In 17 subjects undergoing VV-ECMO who also failed at least one weaning attempt, prolonged prone positioning (24 hours) was performed [65]. Respiratory system compliance increased from 18 (12–36) to 32 (15–36) mL/cmH_2_O (*p* < 0.0001), and the PaO_2_/FiO_2_ ratio increased from 111 (84–128) to 173 (120–203) mmHg (*p* < 0.0001). Similar findings were reported in several case series and observational cohort studies [66–69]. Indications of prone positioning during ECMO include difficult-to-wean, severe hypoxia (PaO_2_/FiO_2_ < 70) and injurious ventilator setting with plateau pressure exceeding 32 cmH_2_O [70].

One challenging issue in performing prone positioning is the potential risk of turning the patient. Thus, some authors propose that ECMO may be a relative contraindication of prone positioning [67]. Reported adverse effects include cannula malfunction, inadvertent extubation, bed sore, and dislodged arterial and central venous lines [71]. Cannula and chest tube site bleedings were also noted in some studies [72, 73]. A standard turning procedure should be protocolized in specialized centers to avoid these potentially detrimental events. There is evidence that prone positioning during ECMO is safe if performed properly [74, 75].

## 7. Spontaneous Breathing during ECMO

Spontaneous breathing is usually not allowed during early phase of severe ARDS, mostly because these critically ill patients require protective ventilation (e.g., low tidal volume, high positive end-expiratory pressure, and recruitment maneuver) [76]. To perform protective ventilation, patients usually require deep sedation and paralysis. In ACURASYS (ARDS et Curarisation Systematique) trial, the use of neuromuscular blocking agents to suppress spontaneous breathing was found to be beneficial on clinical important outcomes such as ICU-free days and mortality (hazard ratio at 90 days: 0.68; 95% CI: 0.48–0.98). The effect was statistically significant in severe ARDS (90-day mortality: 30.8% versus 44.6%, *p* = 0.04) [77]. Similar results have been reported in other studies [78–82]. However, adverse effects of deep sedation and paralysis, including bradycardia, ICU-acquired paresis, ventilator-associated pneumonia, are still important concerns. To avoid potential adverse effects of deep sedation and paralysis, some pioneering centers start to use ECMO as the first line therapy, rather than rescue therapy after MV failure. Thus, there is accumulating evidence on the use of ECMO in awake, spontaneously breathing patients [83–85]. In patients waiting for lung transplantation, those underwent ECMO with spontaneous breathing demonstrated improved survival when compared to other bridging strategies [84].

ECMO may provide an alternative to deliver protective ventilation. As previously mentioned, carbon dioxide removal is able to control spontaneous breathing effort. With more carbon dioxide removal by increasing gas and blood flow, apnea can be induced in animals [17, 18]. Similar results have been found in human studies [19, 86]. In late phase of severe ARDS, spontaneous breathing can be allowed to prevent adverse impact of long-term controlled ventilation. For example, respiratory muscle atrophy is common in patients with prolonged mechanical ventilation, and the adverse effect can occur at as few as 18 hours after mechanical ventilation [87]. Restoration of respiratory muscle activity is helpful to decrease or prevent such disuse myopathy [88]. Another benefit of spontaneous breathing is its systemic and preportal organ blood flow. In an animal study, Hering and coworkers showed that the stomach blood flow increased from 0.13 ± 0.01 to 0.29 ± 0.05 mL/g·min with spontaneous breathing. Similar trends were found in other visceral organs [89]. It is well known that visceral organ perfusion is an important determinant of clinical outcomes in the critically ill. In a case series of six participants, Karagiannidis and colleagues found that patients could immediately regulate PaCO_2_ towards a physiological range. Tidal volume was increased from 2–5 mL/kg to 8 mL/kg with inactivated ECMO, and inspiratory pressure increased from 19–29 cmH_2_O to 21–45 cmH_2_O [56]. Spontaneous breathing in severe ARDS animals undergoing ECMO support was associated with improved oxygenation and intrapulmonary shunt and redistributed ventilation towards dorsal areas, as compared to those with controlled ventilation [44]. The mechanical ventilation mode allowing for spontaneous breathing can be assisted mode, continuous positive airway pressure plus pressure support, and neural adjusted mechanical ventilation.

Furthermore, allowing spontaneous breathing during ECMO may be beneficial in terms of early rehabilitation, because these patients requires less sedation and paralysis. It is possible to perform early rehabilitation for this group of patients. In a study involving 100 ECMO patients, investigators found that 35% (35/100 patients receiving ECMO) could participate in early mobilization and that 51% (18/35) were able to walk [90]. Thus, early mobilization is considered safe and feasible. There is evidence that patients receiving physical training can have much shorter duration of ICU stay [91].

In aggregate, spontaneous breathing is not allowed at early phase of severe ARDS, aiming to perform protective ventilation. With ECMO support, there is no worrisome on hypoxemia and hypercapnia and protective ventilation can be easily delivered. At recovery phase of severe ARDS, it may be wise to lower the ECMO sweep gas and blood flows, allowing recovery of spontaneous breathing. The recovery can be very quick.

## 8. Weaning

Some authors proposed that weaning VV-ECMO should start with ventilator weaning. The procedure may begin when the patient was able to maintain adequate gas exchange with decreasing ECMO and sweep flow and minimal ventilator setting. Patients can be weaned from mechanical ventilation while still on ECMO therapy. The use of single-site, dual lumen catheter in the internal jugular vein allows extubated patients to be ambulatory while being connected to the ECMO circuit. Such a strategy requires a good teamwork among nurses, physicians, and other medical workers [92]. Thereafter, when the FiO_2_ is weaned on ECMO, the flow rate can be decreased below 2.5 L/min. Decannulation can be considered when the patient is treated at lowest FiO_2_ and ECMO flow.

Other authors prefer the use of a lung-protective MV approach and later decide to prioritize weaning VV-ECMO over MV [47]. In an international survey involving 141 individual responses, Marhong and colleagues reported that the majority of centers prioritized weaning VV-ECMO over mechanical ventilation [46]. The weaning protocol can be performed as recommended by extracorporeal life support organization (ELSO) guidelines (https://www.elso.org): ECMO flows are decreased in steps to a minimum of 1 L/min while maintaining sweep at 100%. Alternatively, the flows are decreased to 2 L/min and then the sweep FiO_2_ is decreased. Both approaches should aim to maintain SaO_2_ greater than 95%. When SaO_2_ is stable on this setting, the sweep can be clamped on ventilator settings of pressure support ventilation (PSV) or continuous positive airway pressure (CPAP) of 20 cmH_2_O. If SaO_2_ > 95% and PaCO_2_ < 50 mmHg can be maintained for 60 minutes, ECMO can be weaned.

## 9. Conclusions

Although MV is commonly employed to avert catastrophic hypoxemia and hypercapnia in patients with severe ARDS, MV per se can cause lung injury and accelerate the disease progression. Extracorporeal membrane oxygenation (ECMO) provides an alternative to rescue patients with severe respiratory failure that MV fails to maintain adequate gas exchange. The timing of ECMO initiation based on the risks and benefits of ECMO has been widely investigated. In the running of ECMO, the protective ventilation strategy can be employed without worrying about catastrophic hypoxemia and carbon dioxide retention. There is a large body of evidence showing that protective ventilation with low tidal volume, high PEEP, and prone positioning can provide benefits on mortality outcome. More recently, there is an increasing popularity on the use of awake and spontaneous breathing for patients undergoing ECMO. Lastly, we discussed ECMO weaning. The majority of centers prioritized weaning VV-ECMO over mechanical ventilation, while others preferred to wean MV first.

## Figures and Tables

**Table 1:** Sliding scale of esophageal pressure-guided titration of PEEP. The table was adapted from [59]. Ventilator setting is adjusted in one column at a time to keep the partial pressure of arterial oxygen (PaO_2_) between 55 and 120 mmHg. Alternatively, the oxygen saturation, as measured by pulse oximeter, is kept between 88 and 98% by using the ventilator settings in one column at a time. The positive end-expiratory pressure (PEEP) is set at such a level that transpulmonary pressure during end-expiratory occlusion (PLexp) stays between 0 and 10 cmH_2_O and keeps transpulmonary pressure during end-inspiratory occlusion at less than 25 cmH_2_O.

FiO_2_	0.4	0.5	0.5	0.6	0.6	0.7	0.7	0.8	0.8	0.9	0.9	1.0
Plexp	0	0	2	2	4	4	6	6	8	8	10	10

**Table 2:** Sliding scale of PEEP/FiO_2_ combinations to maintain oxygenation. Positive end-expiratory pressure (PEEP) represents the level set at ventilator and not levels of total PEEP, auto-PEEP, or intrinsic PEEP.

FiO_2_	0.3	0.4	0.4	0.5	0.5	0.6	0.7	0.7	0.7	0.8	0.9	0.9	0.9	1.0	1.0
PEEP	5	5	8	8	10	10	10	12	14	14	14	16	18	18	20–24
